# Terahertz Artificial Dielectric Lens

**DOI:** 10.1038/srep23023

**Published:** 2016-03-14

**Authors:** Rajind Mendis, Masaya Nagai, Yiqiu Wang, Nicholas Karl, Daniel M. Mittleman

**Affiliations:** 1 Brown University, School of Engineering, Providence, RI 02912, USA; 2 Osaka University, Graduate School of Engineering Science, Toyonaka Osaka 560-8531, Japan; 3 Rice University, Department of Electrical and Computer Engineering, Houston, TX 77005, USA

## Abstract

We have designed, fabricated, and experimentally characterized a lens for the THz regime based on artificial dielectrics. These are man-made media that mimic properties of naturally occurring dielectric media, or even manifest properties that cannot generally occur in nature. For example, the well-known dielectric property, the refractive index, which usually has a value greater than unity, can have a value less than unity in an artificial dielectric. For our lens, the artificial-dielectric medium is made up of a parallel stack of 100 μm thick metal plates that form an array of parallel-plate waveguides. The convergent lens has a plano-concave geometry, in contrast to conventional dielectric lenses. Our results demonstrate that this lens is capable of focusing a 2 cm diameter beam to a spot size of 4 mm, at the design frequency of 0.17 THz. The results further demonstrate that the overall power transmission of the lens can be better than certain conventional dielectric lenses commonly used in the THz regime. Intriguingly, we also observe that under certain conditions, the lens boundary demarcated by the discontinuous plate edges actually resembles a smooth continuous surface. These results highlight the importance of this artificial-dielectric technology for the development of future THz-wave devices.

Artificial dielectrics are man-made media that mimic the properties of naturally occurring dielectric media, or even manifest properties that cannot generally occur in nature. For example, the well-known dielectric property, the refractive index, which usually has a value greater than unity, can have a value less than unity in an artificial dielectric. This idea was first, briefly introduced by the microwave community over half a century ago^1^,^2^,^3^. Fascinatingly, the wavelength scaling that arises when transitioning from the microwave regime to the terahertz (THz) regime gives new life to these artificial media by allowing more practicable and manageable structural dimensions^4^,^5^,^6^,^7^,^8^,^9^,^10^. In this work, we experimentally demonstrate and characterize a convergent lens designed using this artificial-dielectric concept, suitable for the THz regime. We demonstrate that this artificial dielectric lens can focus THz waves just as a conventional dielectric lens does, and with higher overall transmission than some commonly used dielectric materials.

The artificial medium consists of a stack of metal plates forming a uniform array of parallel-plate waveguides (PPWGs) that operate in tandem. Therefore, without loss of generality, we can understand the operating principle by considering only a single PPWG. The waveguide’s mode of interest is the lowest-order transverse electric (TE_1_) mode, whose electric-field vector is oriented parallel to the plates^11^. Considering the phase velocity of the TE_1_ mode, we can derive an effective refractive index *n*, analogous to a conventional dielectric^4^. It follows that *n* is frequency dependent such that 0 ≤ *n* < 1, where the equality holds at the mode’s cutoff frequency. This unique frequency dependence is shown in the inset of Fig. 1(d), for a 1 mm plate separation, corresponding to a cutoff of 0.15 THz.

## Device Design and Fabrication

**Figure 1:**
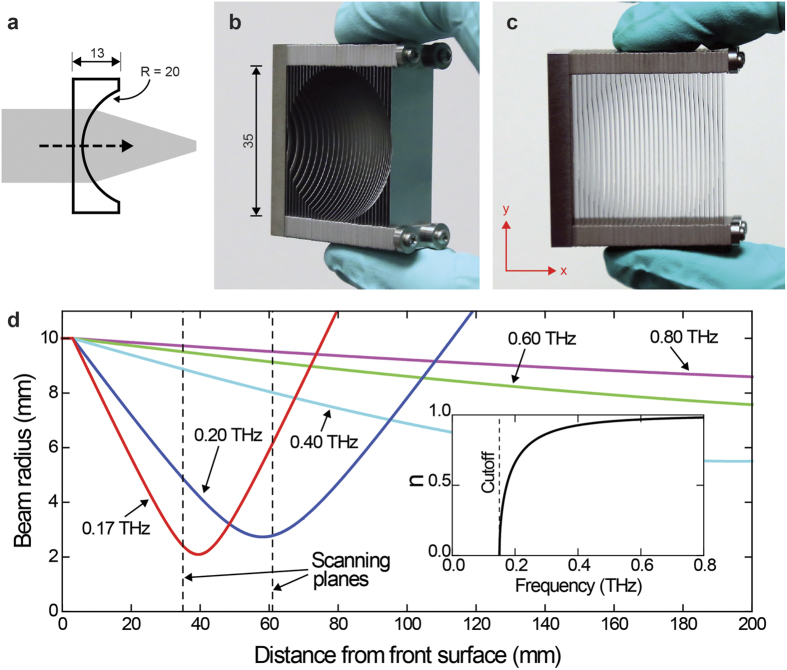
(**a**) Schematic of the plano-concave artificial-dielectric lens. Dimensions are in millimeters. Two photographs of the actual device, looking from the output side, are given in (**b**,**c**). (**d**) Theoretical Gaussian beam analysis of the evolution of the output beam for an input beam diameter of 2 cm, for the frequencies of 0.17, 0.20, 0.40, 0.60, and 0.80 THz. The scanning planes (at 35 mm and 61 mm) employed in the experimental investigation are indicated by the black dashed lines. The inset shows the effective refractive index of the artificial dielectric having a 1 mm plate separation, corresponding to a cutoff of 0.15 THz.

Since *n* is less than that of empty space, we need to employ a concave geometry to realize a convergent (or focusing) lens, in contrast to the typical convex geometries employed with conventional dielectrics. Therefore, the lens design resembles a plano-concave geometry as illustrated in Fig. 1(a). The lens consists of a parallel stack of 32 stainless-steel plates of 100 μm thickness, spaced 1 mm apart, and provides a clear aperture of 35 mm. This plate thickness (with a tolerance of 10%) was sufficiently small to prevent undesirable reflection losses, and provided adequate robustness to maintain the required uniform spacing between the plates. Two photographs of the fabricated device are shown in Fig. 1(b,c). In the design, the radius of curvature (on the concave, output side) progressively decreases, going from the inner-most plates to the peripheral ones. Thus, this unique set of plates effectively emulates a spherical, concave surface with a 20 mm radius at the output end and a planar flat surface at the input end. The plates (and spacers) were formed by chemical etching to avoid any strain or burr during fabrication. As seen in the figure, the device was assembled by stacking the plates and spacers intermittently along four locating poles via etched registration holes in the plates.

The fundamental physical mechanism underlying the focusing behavior of a lens is refraction. Therefore, since the effective refractive index of the artificial-dielectric medium is frequency dependent, we can expect the focusing behavior to be frequency dependent as well. This means that both the focused spot size and the focal length will vary with frequency. More specifically, frequencies that are closer to the cutoff of the PPWG will have a tighter focus and a shorter focal length, because the closer the frequency to cutoff, the larger the index difference (in relation to empty space). In order to understand this frequency dependent focusing behavior, we first carried out a theoretical Gaussian beam analysis. The predicted result for the final design is presented in Fig. 1(d) [corresponding to the lens geometry shown in Fig. 1(a)]. This plot shows the evolution of the output beam radius for the frequencies of 0.17, 0.20, 0.40, 0.60, and 0.80 THz. The radius is specified in terms of the (1/*e*)-Gaussian-amplitude, for an assumed input beam radius of 10 mm. This clearly indicates a progressively tighter focus and a shorter focal length as the frequency gets closer to the cutoff of 0.15 THz, as anticipated. In fact, according to theory, very close to the cutoff, at a frequency of 0.17 THz, a 20 mm diameter input beam can be focused to a spot size of 4 mm. For frequencies far away from cutoff, there is virtually no focusing since the index difference becomes negligible.

## Transmission Measurement

**Figure 2:**
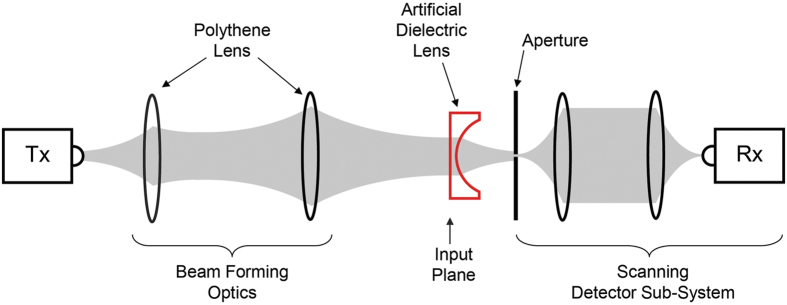
Schematic of the experimental setup used to investigate the transmission characteristics of the artificial-dielectric lens. Tx and Rx are the fiber-coupled THz transmitter and receiver, respectively. The gray-color areas denote the THz beam.

The experimental investigations were carried out using a fiber-coupled THz time-domain-spectroscopy system. This system is capable of generating (and detecting) a train of temporal pulses having a THz baseband. The input beam into the artificial-dielectric lens was formed to a frequency-independent diameter of approximately 20 mm. The THz detection sub-system consisted of a one-to-one confocal imaging system with an effective input aperture of 1 mm diameter to improve the spatial resolution. Signals were detected on square planar grids transverse to the optic axis by scanning the whole sub-system in 0.5 mm (or 1 mm) steps using two motorized steppers. Figure 2 illustrates the schematic of the setup employed to characterize the transmission through the lens, showing the input beam-forming optics and the scanning detector sub-system carrying the aperture. In order to effectively characterize the frequency dependent focusing behavior of the lens, we measured the output beam at two grid planes, one plane 35 mm away from the input plane of the lens, and the other 61 mm away from the input plane. [These two scanning planes are indicated by black dashed lines in Fig. 1(d).] For both of these measurements, we chose a 40 mm × 40 mm grid with a step size of 0.5 mm, to accommodate the expected small spot sizes due to the predicted strong focusing power. As such, a single output-beam measurement resulted in 6561 data points. In addition to the output-beam measurements, we also measured the input beam, by removing the lens and moving the detector sub-system such that the aperture coincided with the input plane of the lens. For this measurement, we chose the same grid size but with a larger, 1 mm step size, since the input beam was formed to be approximately 20 mm in diameter.

**Figure 3:**
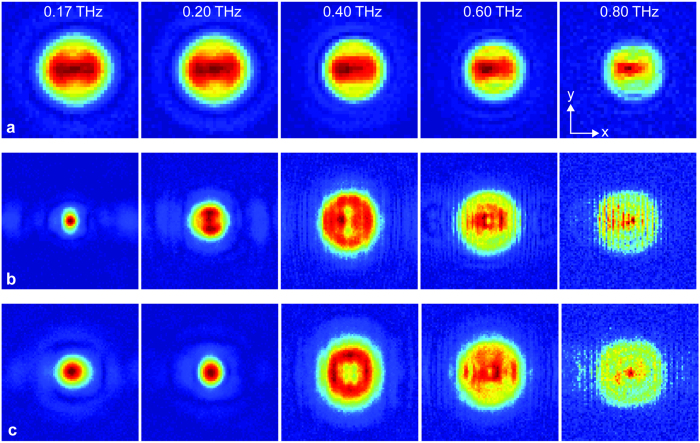
Measured electric field maps of the (**a**) input beam, (**b**) output beam at 35 mm away, and (**c**) output beam at 61 mm away, for the frequencies of 0.17, 0.20, 0.40, 0.60, and 0.80 THz. All are false-color plots.

To analyze the data, each detected time-domain signal was Fourier transformed to extract the spectral amplitude. These data were then used to construct the transverse electric field profiles of the propagating beam at various frequencies. The false-color 2D plots of these electric field profiles are shown in Fig. 3, for the frequencies of 0.17, 0.20, 0.40, 0.60, and 0.80 THz, similar to that investigated theoretically. Each square plot is of size 40 mm. The horizontal panel in Fig. 3(a) corresponds to the input beam, and exhibits a more or less frequency independent overall beam size, as expected from the input beam forming optical configuration. There is a mild decreasing trend with increasing frequency, which can be attributed to the usual frequency dependent diffraction. The panels in Fig. 3(b,c) correspond to the output beams at the 35 mm and 61 mm scanning planes, respectively. The smallest focal spot is observed in Fig. 3(b), in the 35 mm scanning plane, and occurs at a frequency of 0.17 THz. This is consistent with the location of the beam-waist for this frequency, as predicted by the Gaussian-beam analysis shown in Fig. 1(d). Beyond this frequency, still within the 35 mm scanning plane, the spot size becomes slightly larger at 0.20 THz, and plateaus to a size very close to the input beam size at the higher frequencies. For the panel in Fig. 3(c) corresponding to the 61 mm scanning plane, the smallest beam size is observed for the frequency of 0.20 THz. The beam size is slightly larger at 0.17 THz, and as before, plateaus to an overall size similar to the input beam size at the higher frequencies of 0.40, 0.60, and 0.80 THz. All of these qualitative observations are well in agreement with the results of the theoretical Gaussian-beam analysis shown in Fig. 1(d). Interestingly, we are even able to discriminate the output beam into vertical slices at 0.80 THz (corresponding to the sandwiched beam between the vertically oriented plates of the lens), as a result of the relatively smaller wavelength. These slices are more pronounced at the 35 mm scanning plane due to the close proximity to the lens, thereby undergoing less diffractive overlapping.

**Figure 4:**
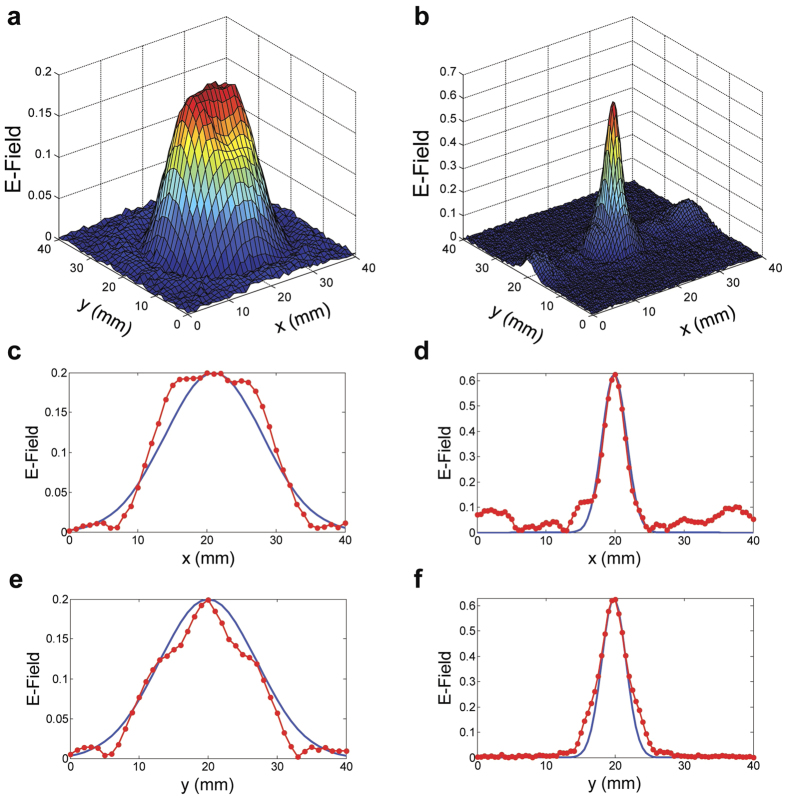
Measured electric field maps of the (**a**) input beam and (**b**) output beam at 35 mm away, for the design frequency of 0.17 THz, in terms of false-color surface plots. The corresponding *x* and *y* cross-sectional slices are given in (**c**,**e**) for the input, and in (**d**,**f**) for the output. The theoretical Gaussian curves (in blue) are directly related to the analytical curves in Fig. 1(d).

Figure 4(a,b) give the false-color 3D surface plots of the input and output electric field profiles at 0.17 THz, respectively. (The output corresponds to the 35 mm plane.) Their cross-sectional slices along with theoretical Gaussian curves are plotted in Fig. 4(c,e) for the input, and in Fig. 4(d,f) for the output. These plots clearly demonstrate the strong focusing effect of the lens, where the 20 mm diameter input beam has been focused to a size of about 5 mm, based on the 1/*e* sizes of the Gaussian comparisons which are directly related to Fig. 1(d). We should point out that, although experimentally we measure only a 5 mm spot due to the 35 mm location of the scanning plane, the theory predicts a smaller (4 mm) spot that is located at 40 mm. Furthermore, the enhancement in the peak electric field is approximately three times, corresponding to an enhancement of nine times in energy density. This enhancement already implies a low insertion loss for the artificial dielectric lens, not surprising due to the inherently low propagation loss of the TE_1_ mode in the THz frequency region^12^,^13^. In the output beam, we observe a non-zero background along the *x* direction, whereas there is no such background along the *y* direction. Incidentally, the *x* direction happens to be the direction perpendicular to the plates, while *y* is the direction parallel to the plates. This implies that there is some imperfection in the focusing along the direction perpendicular to the plates, which is possibly due to the spatially discrete nature of the plate-edges that are assumed to mimic a continuous, concave surface. Nevertheless, our estimates indicate that almost 90% of the THz energy is concentrated within the focal spot.

## Reflection Measurement

**Figure 5:**
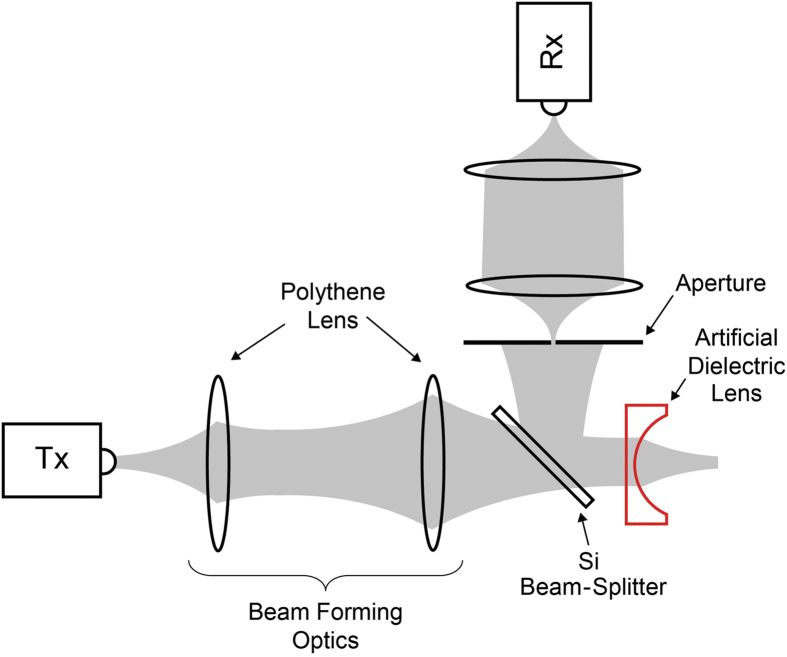
Schematic of the experimental setup used to investigate the reflection characteristics of the artificial-dielectric lens. The aperture was located a total axial path length (with a 90° bend) of 12 cm away from the input plane of the lens.

For a complete understanding of the lens behavior, we also investigated the reflection losses caused by the impedance mismatch. Figure 5 illustrates the schematic of the experimental setup employed to characterize the reflection off of the front surface of the lens. Here, a 45° silicon beam splitter was used to divert part of the on-axis reflection towards the detector sub-system that was now oriented at 90° to the input beam axis. The scanning (aperture) plane was located a total axial path length of 12 cm away from the input plane of the lens. We chose a larger, 50 mm × 50 mm grid size, compared to the transmission measurement, to be able to accommodate the larger reflected (and diffracted) beam size, and a step size of 1 mm. In order to simplify the reflection analysis, a reference measurement was also obtained by removing the lens and situating a polished aluminum disk to coincide with the input plane of the lens. The scanning plane (as well as the grid size and step size) remained unchanged for this measurement. Since aluminum can be considered to be a perfect reflector for THz waves, this measurement essentially characterizes the normally incident beam on the lens.

**Figure 6:**
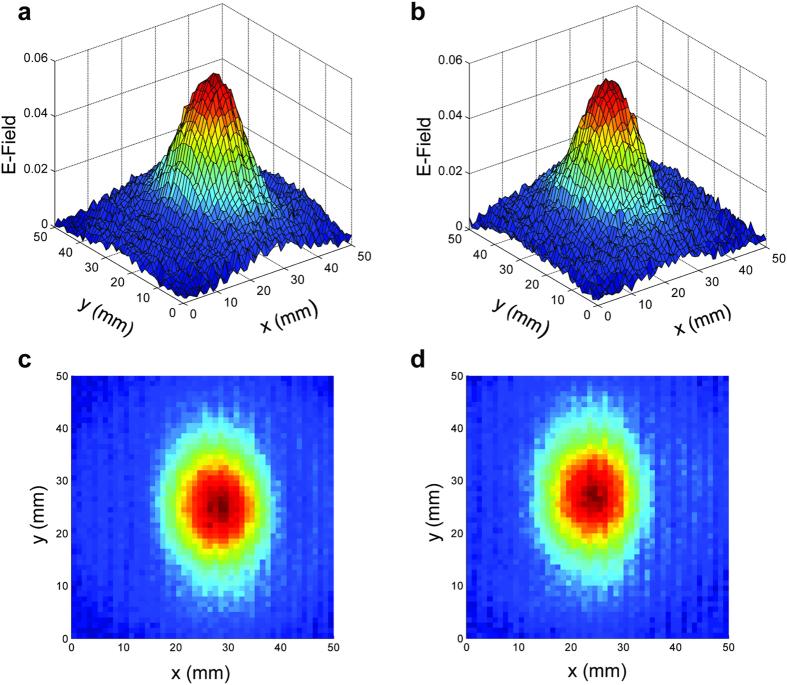
Measured electric field maps of the reflected beam from the (reference) aluminum disk (**a**,**c**) and from the artificial-dielectric lens (**b**,**d**), for the frequency of 0.13 THz.

Similar to the previous investigation, Fig. 6 gives the 3D surface plots and their corresponding 2D plots of the measured electric field amplitude at a frequency of 0.13 THz. This frequency is slightly below the cutoff frequency of 0.15 THz, and as such, we expect all the incident radiation to be reflected without entering the lens. Figure 6(a,c) represents the field map of the aluminum disk, while Fig. 6(b,d) represents that of the lens. We observe that the reflected beams are almost identical (in both amplitude and shape) to each other. In fact, since the scanning plane is axially 12 cm away from the front surface of the lens, this proves that the reflection off of the lens constitutes a well-defined beam. This is despite the fact that the front surface is made up of a series of discrete 100 μm plate edges that are 1 mm apart. This is an intriguing result and implies that below cutoff, the front surface of the stacked-plate array emulates a well-defined solid planar surface, without causing any noticeable scattering effects due to the plate edges. The slight offset seen in the beam centers can be attributed to a slight angular tilt in the disk surface compared to that of the lens.

## Device Performance

**Figure 7:**
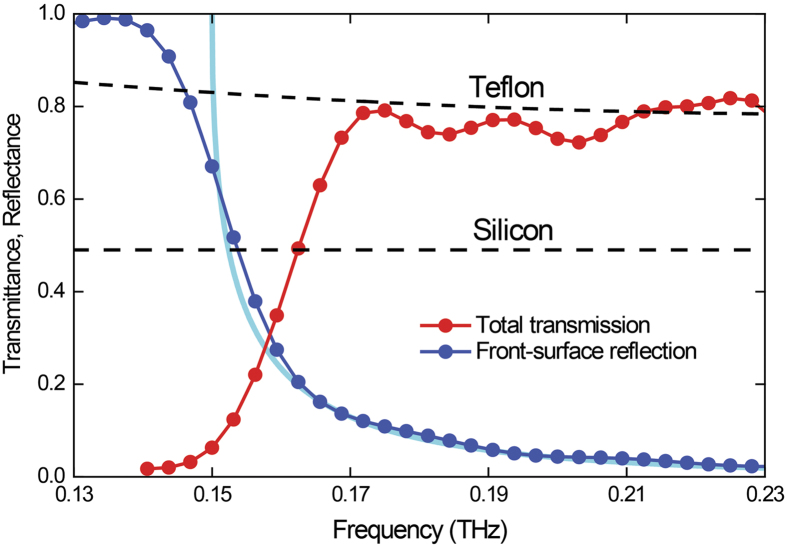
Experimental total transmission (red curve) and front surface reflection (blue curve) for the artificial-dielectric lens. The theoretical front surface reflection is given by the light-blue curve. The black dashed lines give the estimated transmission for similarly convergent lenses made of high-resistivity silicon and Teflon.

Using these electric-field maps, we can deduce the power throughput of the device and the power-reflection losses. These experimentally derived transmittance and reflectance values are plotted as a function of frequency in Fig. 7 by the red and blue curves, respectively. Based on these plots, we can arrive at several important conclusions about the operational behavior of the artificial-dielectric lens. Firstly, there is virtually no power throughput below about 0.14 THz, which is consistent with the TE_1_ mode cutoff of 0.15 THz for a plate separation of 1 mm that was chosen for the design. We can easily shift the cutoff to any THz frequency by altering the plate separation. For comparison, we have also plotted the theoretical reflectance for the front surface of the lens (light-blue curve) in Fig. 7, taking into account the frequency-dependent impedance mismatch. This shows excellent agreement with the experimental curve, except very near to the cutoff, where the finite propagation length of the waveguides becomes relevant.

Secondly, at the design frequency of 0.17 THz (and beyond), the power throughput is almost 80%, signifying a very low insertion loss. We can compare this power throughput with those of conventional THz lenses. High-resistivity (>10 k Ω-cm) silicon and Teflon are two of the most common materials used to fabricate THz lenses. Based on literature data^14^,^15^, we have estimated the power throughput for similarly convergent lenses made of silicon and Teflon, and these estimates are indicated by the black dashed lines in Fig. 7. In the case of silicon, the transmittance is entirely determined by its relatively high refractive index since the material is virtually transparent for THz waves. In the case of Teflon, the transmittance is determined by the combination of both the material absorption and the refractive index. This comparison reveals that the power throughput of the artificial-dielectric lens is either comparable to or better than conventional lenses. This high power throughput of the artificial-dielectric lens results from the extremely low propagation losses associated with the TE_1_ mode of the PPWG^12^,^13^. The power loss is almost entirely due to the impedance mismatch at the input and output surfaces. It is also interesting to compare the power throughput of our artificial-dielectric lens with that of metamaterial-based flat lenses, known as metasurface lenses. A purely theoretical study on a similarly convergent lens for the THz regime has demonstrated a transmittance of 75%^16^, while another experimental study has demonstrated a value of only 20%^17^. Incidentally, we note that there has been a previous THz study on a lens architecture similar to our device, although without any supporting experimental proof^18^.

In conclusion, we have designed and experimentally characterized an artificial-dielectric convergent lens suitable for the THz regime. Our results demonstrate that this lens is capable of focusing a 2 cm diameter beam to a spot size of 4 mm, at the design frequency of 0.17 THz. The results further demonstrate that the power throughput of the lens can be better than conventional lenses made of high-resistivity silicon, and comparable to that made of Teflon. Incidentally, since both the focal spot and focal length are frequency dependent, these lenses may also be exploited for THz multispectral imaging applications^19^. Finally, this work highlights the importance of this artificial-dielectric technology for the development of future THz-wave devices.

## Additional Information

**How to cite this article**: Mendis, R. *et al.* Terahertz Artificial Dielectric Lens. *Sci. Rep.* **6**, 23023; doi: 10.1038/srep23023 (2016).
